# The Human Phospholipase B-II Precursor (HPLBII-P) in Urine as a Novel Biomarker of Glomerular Activity in COVID-19 and Diabetes Mellitus

**DOI:** 10.3390/jcm13092540

**Published:** 2024-04-26

**Authors:** Shengyuan Xu, Michael Hultström, Anders Larsson, Miklos Lipcsey, Cecilia Lindskog, Sara Bülow, Robert Frithiof, Per Venge

**Affiliations:** 1 Department of Medical Sciences, Clinical Chemistry, Uppsala University, SE-751 85 Uppsala, Sweden; shengyuan.xu@medsci.uu.se (S.X.); anders.larsson@akademiska.se (A.L.); 2 Diagnostics Development a P&M Venge Company, SE-753 12 Uppsala, Sweden; 3 Department of Medical Cell Biology, Integrative Physiology, Uppsala University, SE-751 23 Uppsala, Sweden; michael.hultstrom@mcb.uu.se; 4 Department of Surgical Sciences, Anaesthesiology and Intensive Care, Uppsala University, SE-751 85 Uppsala, Sweden; miklos.lipcsey@uu.se (M.L.); sara.bulow@uu.se (S.B.); robert.frithiof@uu.se (R.F.); 5 Hedenstierna Laboratory, Department of Surgical Sciences, Uppsala University, SE-751 85 Uppsala, Sweden; 6 Department of Immunology, Genetics and Pathology, Uppsala University; SE-751 23 Uppsala, Sweden; cecilia.lindskog@igp.uu.se

**Keywords:** acute kidney injury (AKI), lipocalin, KIM-1, NGAL, cystatin C

## Abstract

**Background:** The human phospholipase B-II precursor (HPLBII-P) was originally purified from white blood cells but is also found in other cellular structures, such as kidney glomeruli and tubuli. The objective of this report was to investigate the relationship of HPLBII-P in urine to acute kidney injury in patients with COVID-19. **Methods:** Urine was collected at admission from 132 patients with COVID-19 admitted to the intensive care units (ICUs) because of respiratory failure. HPLBII-P was measured using a sensitive ELISA. For comparison, human neutrophil lipocalin (HNL) was measured in urine, using the ELISA configured with the monoclonal antibody 763/8F, as a sign of tubular affection in addition to routine biomarkers of kidney disease. **Results:** Overall, the concentrations of urinary HPLBII-P were almost 3-fold higher in patients with COVID-19 compared to healthy controls (*p* < 0.0001) and with significantly higher concentrations even in patients with COVID-19 without signs of acute kidney injury (AKI) (*p* < 0.001). HPLBII-P was further increased in patients with AKI (*p* < 0.02). HPLBII-P was significantly increased in patients with diabetes mellitus (*p* = 0.0008) and correlated to plasma glucose (r = 0.29, *p* = 0.001) and urine albumin concentrations (r = 0.55, *p* < 0.001). **Conclusions:** Urine concentrations of HPLBII-P are highly raised in the urine of patients with COVID-19 and relate to AKI and diabetes mellitus. HPLBII-P may reflect glomerular injury and/or increased glomerular cell activity in SARS-CoV-2 infections.

## 1. Introduction

The human phospholipase B precursor (HPLB-P) was originally purified from white blood cells obtained from healthy human blood donors and was, at that time, only known as a hypothetical protein based on a sequence analysis of the human genome [1]. Subsequently, we identified the nature of this protein since it displayed phospholipase activities with the ability to remove fatty acids from both sn-1 and sn-2 bonds in phospholipids, thus potentially giving rise to a broad panel of active fatty acid molecules when released from its cellular origin.

By means of our specific polyclonal antibodies, most human organ tissues were screened by immunohistochemistry for the expression of HPLBII-P [2]. Three organ tissues expressed the protein particularly well. These were neuronal cells, gastrointestinal and kidney tissues, and bone marrow cells. By means of ELISA, we measured HPLBII-P in gastrointestinal material and found interesting relations to GI diseases such as Irritable Bowel Syndrome (IBS) [3] and Inflammatory Bowel Disease (IBD) [2]. Our assay also allows the measurement of the very low concentrations found in urine, as shown in this report. The preliminary findings of the measurement of HPLBII-P in urine suggested that this biomarker might be a sensitive indicator of acute kidney injury (AKI). The purpose of this report was to evaluate the potential utility of HPLBII-P as a sign of kidney injury and compare its potential with other currently used and well-characterised biomarkers of tubular and glomerular injury such as Cystatin C in urine and plasma [4], urine excretion of albumin, KIM-1 (Kidney Injury Molecule-1) [5], HNL/NGAL (Human Neutrophil Lipocalin/Neutrophil Gelatinase Associated Lipocalin) [6,7], and TIMP-2 (Tissue inhibitor of metalloproteinases 2) [8]. The early identification of AKI in patients with COVID-19 infection is important and may be associated with a high risk of developing serious organ failures [9]. Thus, we collected urine from a cohort of critically ill patients with COVID-19 admitted to the ICU because of respiratory failure and in whom AKI was a common complication of the infection.

## 2. Material and Methods

Data in this research are part of the PronMed study. This study was approved by the National Ethical Review Agency (Dnr 2017/043, with amendments 2020-01623, 2020-02719, 2020-05730, 2021-01469, and 2022-00526-01) and listed at ClinicalTrials.gov (NCT03720860). Informed consent was obtained from the patient or next of kin. The Declaration of Helsinki and its subsequent revisions were followed.

One hundred and thirty-two patients were admitted to the ICU (intensive care unit) of Uppsala University Hospital with SARS-CoV-2 infections as diagnosed with PCR and signs of organ failure. Detailed information of the patient demographics was given previously [10,11]. The healthy group consisted of 40 women (median age 32 years, range 22–64 years) and 18 men (median age 26 years, range 22–63 years).

Clinical data were collected from the electronic medical records and AKI severity was staged according to kidney disease: Improving Global Out come (KDIGO) creatinine criteria and renal replacement requirement solely [12].

Immunohistochemistry of normal kidney tissue was performed using the rabbit polyclonal antibody raised against HPLBII-P. The procedure of tissue sampling and staining procedures are documented in the Human Protein Atlas (https://www.proteinatlas.org/, accessed on 8 February 2024).

**Figure 1:**
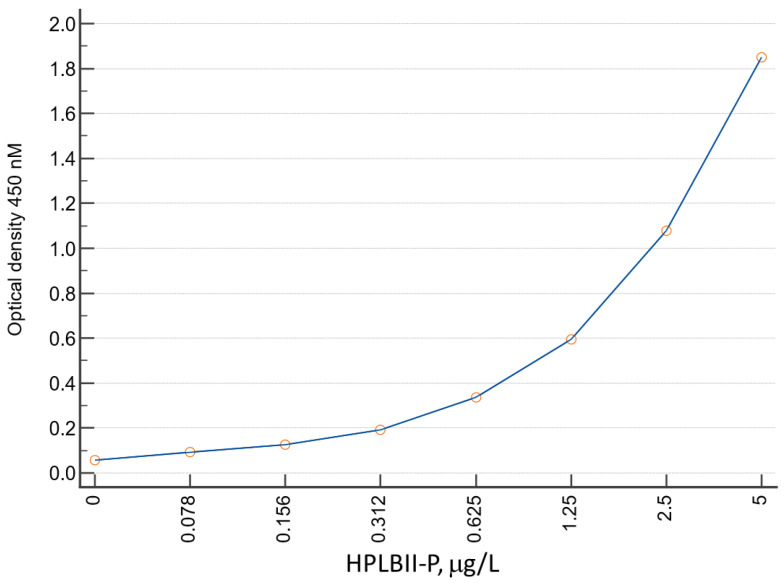
The figure shows a representative standard curve with the concentrations of HPLBII-P on the x-axis and the optical density at 450 nm on the y-axis.

HPLBII-P was measured using a novel ELISA. A polyclonal antibody–based ELISA was developed in this study. Briefly, microtiter plates (Nunc Maxsorp, Merck, Darmstadt, Germany) were coated with rabbit anti-PLB-P polyclonal antibody (100 μL/well, 2.5 μg/mL) diluted in carbonate–bicarbonate buffer (0.05 mol/L Na_2_CO_3_–NaHCO_3_, pH 9.6) (Invitrogen Corporation, ThermoFisher Scientific, Waltham, MA, USA) at 4 °C overnight. Additional binding sites were blocked with StabilCoat Immunoassay Stabilizer (Surmodics IVD, Inc. Eden Prairie, MN, USA), at room temperature for 40–60 min. We then added 100 μL standards (0.078 to 5 ng/mL, in duplicates) or samples diluted in assay solution (PBS containing 0.2% BSA, 0.1% Tween-20, 0.05% cetrimonium bromide and 0.02% NaN_3_) and incubated the mixtures at room temperature for 1.5 h. Subsequently, 100 μL of diluted biotinylated rabbit polyclonal antibody against human HPLBII-P was added to each well and incubated at room temperature for 60 min, followed by the addition of 100 μL/well of diluted streptavidin-conjugated horseradish peroxidase (GE Healthcare, Uppsala, Sweden) in StabilZyme HRP Conjugate Stabilizer Surmodics IVD, Inc., incubated for 30 min at room temperature. The plates were washed 4 times in a washing buffer (PBS containing 0.05% Tween-20) with a microplate washer (Anthos Fluido, Eugendorf, Austria) between all steps. The enzyme reaction was visualized by use of a 3,3′,5,5′-tetramethylbenzidine solution (100 μL/well) (Sigma-Aldrich, Merck, Darmstadt, Germany) as substrate at room temperature for 15 min, and the reaction was stopped by adding 1 mol/L H_2_SO_4_ (100 μL/well). Absorbance was measured at 450 nm with a reference reading at 540 nm in blank wells using a microplate reader (SPECTRAmax 250, GMI, Molecular Devices, LLC, San Jose, CA, USA). A representative standard curve is shown in Figure 1. The CV% of duplicate standards varied between 0.1 and 2.0. With-in assay imprecision was 3.2% (*n* = 7). LOD (Level of Detection) was <0.05 μg/L. Lowest reported concentration in urine was 0.1 μg/L. The urine was diluted 5 times in assay buffer. The stability of HPLBII-P in biological samples was shown previously [1,2]. No change in concentrations was seen after 5 freeze–thaw cycles.

The HNL ELISA (Diagnostics Development, Uppsala, Sweden) was configured with mabs 763 and 8F for the purpose of catching most HNL molecular variants in urine. The analytical performance of the HNL (763/8F) ELISA of duplicate samples was <10% CV (Coefficient of Variation) of duplicate samples. The measurements of HPLBII-P and HNL (763/8F) were performed on all samples collected consecutively between 2021 and 2022. The measurements of KIM-1, TIMP-2, NGAL were performed by the ELISA kits DY1750B, DY971, and DY1757, respectively, all purchased from R&D systems, Minneapolis, MN, USA. These measurements of TIMP-2 and NGAL were performed on 48 samples collected consecutively during 2021. All clinical data were blinded to the analysing personal.

Albumin and Cystatin C in urine and Cystatin C, creatinine, and glucose in plasma were all measured by the clinical chemistry laboratory at University Hospital, Uppsala, Sweden. As indicated, all samples were obtained at admission and on non-fasting patients.

### Statistics

All data were from results obtained at admission to the ICU. Non-parametric statistics was applied. For comparison between the independent results, the Mann–Whitney U test was used. For the comparison of the results of multiple groups, Kruskal–Wallis ANOVA was applied. Correlations between biomarkers were calculated by Spearman rank correlations. The statistical programme Medcalc was used in all calculations: MedCalc^®^ Statistical Software version 22.016 (MedCalc Software Ltd., Ostend, Belgium; https://www.medcalc.org).

## 3. Results

### 3.1. HPLBII-P in the Kidney

**Figure 2:**
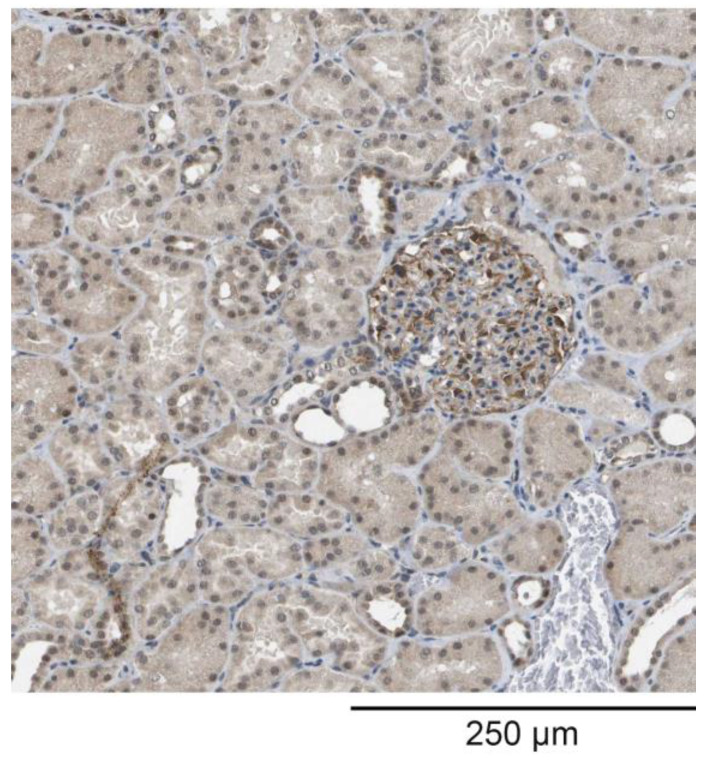
Immunohistochemistry staining of kidney tissue from healthy subjects. Polyclonal antibodies against HPLBII-P were used. The figure shows distinct staining of glomerular cells presumably podocytes and also faint staining of some tubular cells. The figure was generated with permission from the Human Protein Atlas (www.proteinatlas.org, accessed on 8 February 2024).

The staining of kidney tissue with the polyclonal antibodies raised against HPLB-P showed the distinct staining of cells in the glomeruli (Figure 2). Staining was also seen in some tubular cells.

### 3.2. HPLBII-P in the Urine of Patients with COVID-19 

**Figure 3:**
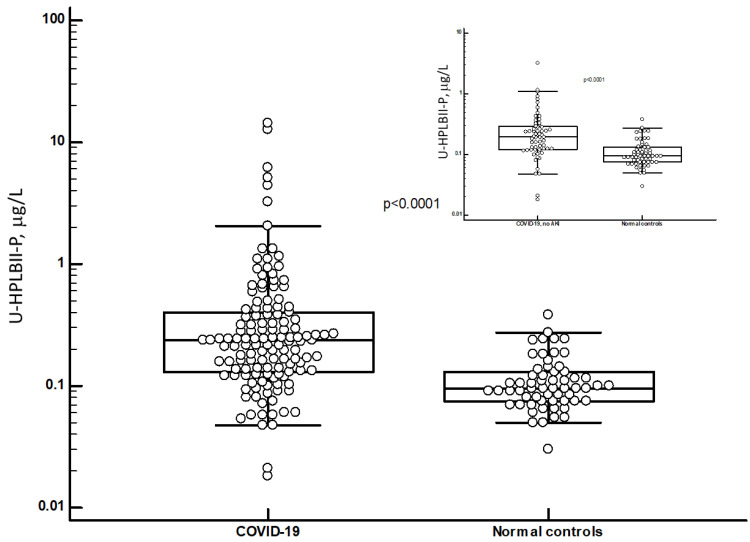
The concentrations of HPLBII-P in urine of patients with COVID-19 and healthy controls. The statistical difference between the groups was evaluated by Mann–Whitney U test, and the significance is shown in the figure. The insert shows the results of patients with COVID-19 without signs of AKI in comparison to healthy controls.

**Figure 4:**
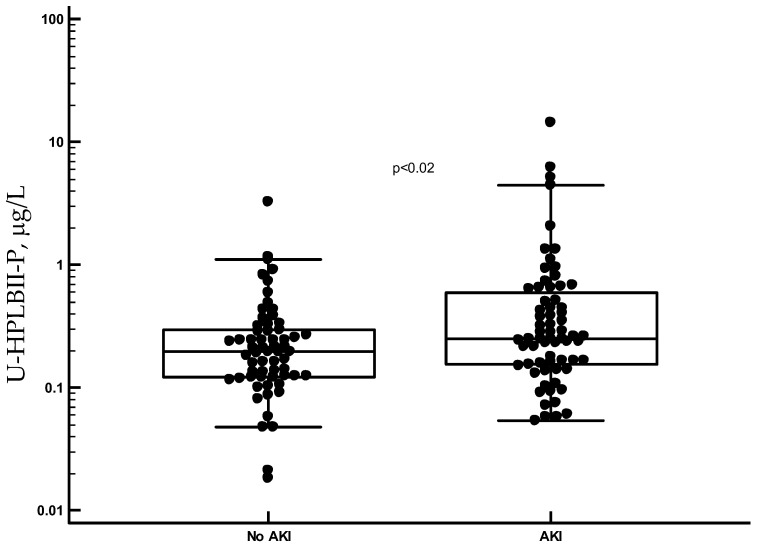
The HPLBII-P concentrations in urine of patients with COVID-19 with and without AKI. The difference of the groups was evaluated by the Mann–Whitney U test, and the significance is shown in the figure.

By means of the HPLBII-P ELISA, the concentrations of HPLBII-P were measured in urine of 58 healthy subjects and of patients with COVID-19. The patients with COVID-19 were all admitted to the ICU because of respiratory failure. It is shown in Figure 3 that the urine concentrations in samples from patients with COVID-19 were highly and significantly increased compared to the results of healthy subjects (*p* < 0.0001). This was also the case when we only looked at patients with COVID-19 without any sign of AKI (as shown in Figure 3). It is further indicated in Figure 4 and Table 1 that the concentrations were higher in the patients with COVID-19 with AKI (*p* < 0.02) and related to AKI stages with (Supplementary Figure S1) or without correction for urine creatinine (*p* = 0.04, Kruskal–Wallis ANOVA).

**Figure 5:**
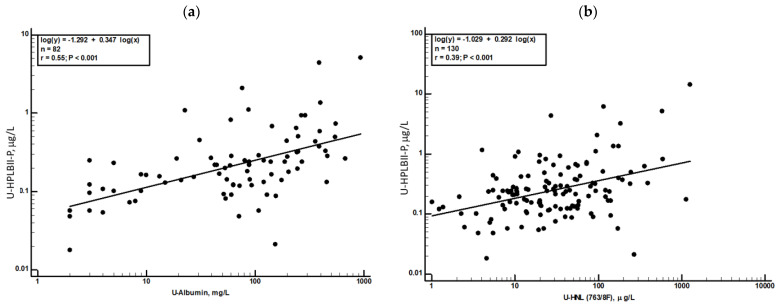
Regression analysis of the correlations between urine concentrations of HPLBII-P and urine Albumin (panel **a**) and urine HNL (763/8F) (panel **b**). The regression equations and r-values are shown in the figures.

In Table 1, we show the concentrations of several other biomarkers in urine in patients with COVID-19 with or without AKI. None of the biomarkers were elevated in patients with AKI, whereas the serum concentrations of Cystatin C were significantly higher in those patients with AKI (*p* = 0.0002). After urine creatinine correction, the levels of HPLBII-P became even higher in AKI relative to No AKI (*p* = 0.002) (Table 2) (supplementary Figure S1) and to AKI stages (Supplementary Figure S1). The relationships of these biomarkers to HPLBII-P were evaluated by Spearman rank correlation analysis. Significant correlations were found with albumin and HNL (763/8F) in urine, as illustrated in Figure 5a,b. A correlation was also found to KIM-1 (r_s_ = 0.28, *p* = 0.01).

### 3.3. HPLBII-P in patients with Diabetes Mellitus

**Figure 6:**
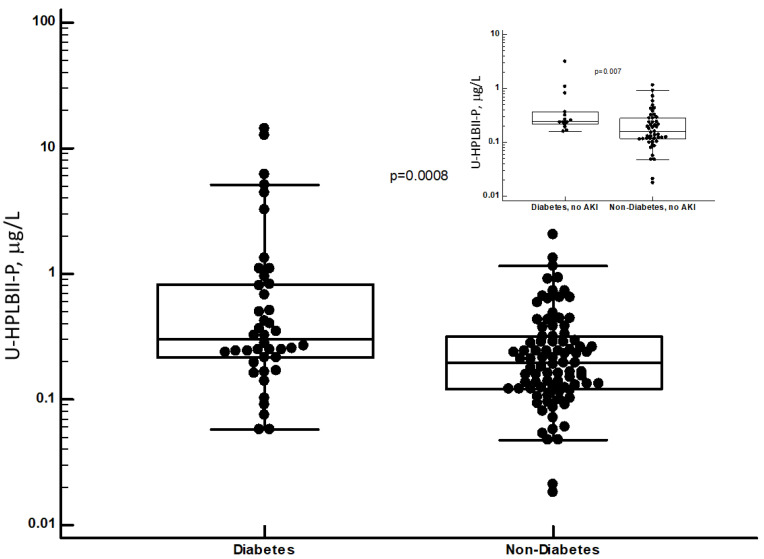
The urine concentrations of HPLBII-P in patients with COVID-19 with or without diabetes mellitus. The difference was evaluated by the Mann–Whitney U test and the significance given in the figure. The insert shows the results in the same cohorts but without AKI.

**Figure 7:**
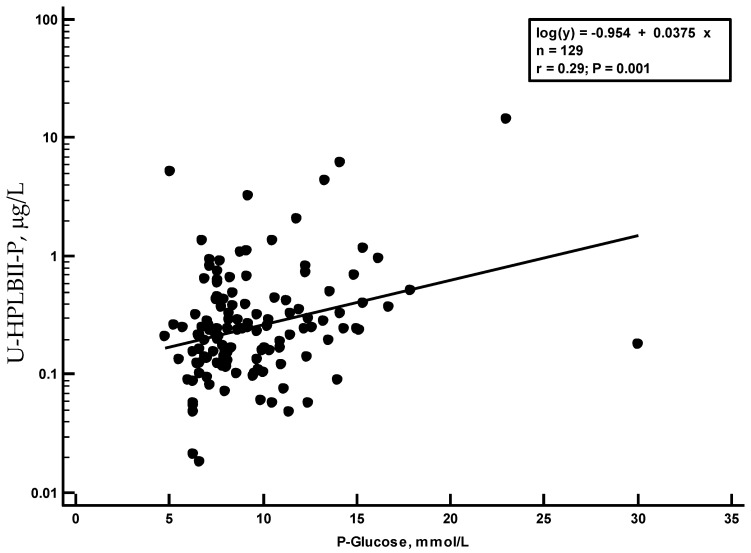
Regression analysis of the correlations between urine concentrations of HPLBII-P and plasma glucose concentrations. The regression equation and r-value are shown in the figure.

In Figure 6, we show the highly and significantly raised concentrations of HPLBII-P in patients with diabetes mellitus (*p* = 0.0008). In Figure 6, we show the increased concentrations in diabetes mellitus in patients with COVID-19 without signs of AKI (*p* = 0.007). Higher concentrations in urine in patients with diabetes mellitus were also seen for HNL (763/8F) (*p* = 0.0056) and NGAL (*p* = 0.007), but not for the other investigated biomarkers shown in Table 1 and Table 2, whether corrected for urine creatinine concentrations or not. After urine creatinine correction, similar results were obtained (Supplementary Figure S2). A significant correlation (r_s_ = 0.29, *p* = 0.001) was seen between the plasma glucose concentrations at admission day and the HPLBII-P concentrations (Figure 7). No correlation to plasma glucose was seen for any of the other urine biomarkers i.e., Albumin, HNL (763/8F), KIM-1, or TIMP-2.

## 4. Discussion

This report has shown that HPLBII-P can be measured in urine at very low concentrations and that these levels are significantly high in patients with SARS-CoV-2 infections admitted to the ICU because of respiratory failure. In our cohort, 61% of the patients had AKI of varying severities, and these patients had higher concentrations of HPLBII-P in their urine than those without AKI, whereas the other investigated biomarkers did not show any difference. We also showed that patients with COVID-19 and diabetes mellitus had raised concentrations of HPLBII-P in their urine. This was seen in the patients with AKI and in the patients without AKI, in contrast to the findings for HNL (763/8F) and NGAL in which raised concentrations were only found in the subpopulation of patients with AKI. NGAL was advocated as an early and sensitive biomarker in the urine of diabetic nephropathy, which is supported by our findings [13,14,15].

The difference between HNL and HPLBII-P in its localization in the kidney is illustrated in the micrograph in Figure 2. It is apparent that HPLBII-P is localized primarily to glomerular cells in addition to some tubular cells, whereas the unique localization of HNL to tubular [11,16,17], but not to glomerular cells, has been well documented. Most likely, HPLBII-P is produced by the podocytes of the glomeruli.

We found a significant correlation to HNL representing tubular damage, which could suggest a tubular origin of HPLBII-P as well. However, the highest correlation was seen for albumin in urine, which indicates that the glomerular origin of HPLBII-P in urine is in line with our immunohistochemistry results shown in Figure 2. Whether the glomerular contribution to the urine presence is a consequence of glomerular damage in COVID-19 or the stimulated secretion from glomerular cells, i.e., podocytes, cannot be judged from the findings in this report. The contribution from a glomerular leakage, though, seems less likely given the molecular size of ca 130 kD of HPLBII-P.

The SARS-CoV-2 virus was found recently in the glomerular cells of patients with COVID-19 [18,19], but we rarely found viruses in urine [20]. One speculation is that the virus acts as a trigger of HPLBII-P secretion from the glomerular cells. This notion has some support in our findings that the concentrations of HPLBII-P in urine in patients with COVID-19 without signs of AKI were significantly raised compared to the healthy controls and as opposed to the other biomarkers studied in this report.

As of today, we do not know much about the actual biological role of HPBLII-P apart from the fact, referred to in the Introduction, that it has a broad phospholipase activity. The activity of Phospholipase A2 (PLA_2_) has been thoroughly investigated in relation to COVID-19, the activity of which is believed to be a key element in the severe inflammatory state seen in many patients infected by SARS-CoV-2 [21]. However, PLA_2_ may also take part in the fight against virus infiltration [22]. Thus, whether the high HPLBII-P concentrations found in urine of our patients with COVID-19 represent kidney damage and/or viral defence remains to be concluded. Indeed, we found previously very high serum levels of HPLBII-P in patients with Influenza A infection without signs of AKI [2].

The most conspicuous finding in this study was the close relation to diabetes mellitus since the concentrations in urine of HPLBII-P were highly elevated and correlated, as opposed to the other studied biomarkers, to the key findings in diabetes mellitus of disturbances in glucose metabolism. This leads to the speculation that HPLBII-P in urine reflects the glomerular damage and function, which is a well-known consequence of poor control of diabetes mellitus [23]. The two tubular biomarkers HNL and NGAL also showed higher concentrations in diabetes mellitus, but only in those patients with signs of AKI. Thus, raised concentrations of HPLBII-P in urine might be a novel and interesting biomarker of glomerular function and activity in diabetes mellitus. Indeed, the integrity of podocytes, which may be the origin of HPLBII-P in urine, has been studied intensively and shown to be affected in patients with diabetes and in those who develop diabetic nephropathy [24,25,26]. As with another podocyte associated protein i.e., Nephrin [27], HPLBII-P in urine could serve as a unique reflection of podocyte function. Thus, our future focus will be on the establishment of HPLBII-P in this regard.

We conclude from this report that the measurement of HPLBII-P in urine is a sensitive means of revealing AKI in patients with COVID-19 and, as such, more sensitive than conventionally used urine biomarkers. Most likely the origin of HPLBII-P in urine is dual i.e., the tubuli, but particularly glomerular cells. Whether our findings can be extended to AKI caused by other aetiologies remain to be shown. HPLBII-P may also be a novel and unique biomarker for the diagnosis and monitoring of glomerular function and activity in patients with diabetes mellitus and possibly add to the plethora of useful clinical biomarkers for the early detection of AKI [28].

## Figures and Tables

**Table 1:** The table shows the results of all investigated biomarkers in urine and Cystatin C in serum of patients with SARS-CoV-2 infections and the comparisons of the concentrations in patients with or without AKI (left) and in patients with or without diabetes mellitus (right). Differences were evaluated by the Mann–Whitney U test, and the results are given in the table. The median concentrations of HPLBII-P in urine of healthy subjects were 0.095 μg/L (95% CI 0.09–0.110 μg/L).

Biomarker Urine	No AKI Median (95% CI)	AKI Median (95% CI)	*p*-Value Mann-Whitney	Non-Diabetes Median (95% CI)	Diabetes Median (95% CI)	*p*-Value Mann-Whitney
HPLBII-P, mg/L	0.19 (0.15–0.24) *n* = 62	0.25 (0.23–0.35) *n* = 67	*p* = 0.019	0.19 (0.15–0.23) *n* = 88	0.28 (0.24–0.44) *n* = 41	*p* = 0.0008
HNL (763/8F), mg/L	25 (20–39) *n* = 61	29 (20–54) *n* = 67	Ns	23 (16–35) *n* = 87	41 (24–99) *n* = 41	*p* = 0.0056
NGAL, mg/L	45 (21–69) *n* = 19	42 (28–85) *n* = 29	Ns	35 (24–53) *n* = 36	112 (38–213) *n* = 12	*p* = 0.007
TIMP-2, ng/L	3.7 (2.5–4.8) *n* = 19	4.4 (2.4–5.9) *n* = 30	Ns	4.2 (2.7–4.9) *n* = 36	4.1 (1.7–7.2) *n* = 13	Ns
KIM-1, ng/L	2.3 (1.6–5.2) *n* = 47	2.6 (1.6–3.1) *n* = 60	Ns	2.5 (1.9–3.2) *n* = 77	2.4 (1.3–4.7) *n* = 30	Ns
Cystatin C, mg/L	0.56 (0.42–0.83) *n* = 47	0.87 (0.58–1.01) *n* = 60	Ns	0.68 (0.53–0.91) *n* = 77	0.76 (0.42–1.46) *n* = 30	Ns
Albumin, mg/L	68 (50–112) *n* = 47	94 (58–130) *n* = 60	Ns	85 (59–130) *n* = 77	71 (42–111) *n* = 30	Ns
**Biomarker** **Serum**	**No AKI** **Median (95% CI)**	**AKI** **Median (95% CI)**	***p*-Value** **Mann-Whitney**	**Non-Diabetes** **Median (95% CI)**	**Diabetes** **Median (95% CI)**	***p*-Value** **Mann-Whitney**
Cystatin C, mg/L	0.98 (0.80–1.05) *n* = 33	1.19 (1.11–1.35) *n* = 47	*p* = 0.0002	1.07 (0.99–1.12) *n* = 58	1.22 (1.06–1.80) *n* = 23	*p* = 0.055

Ns = non-significant.

**Table 2:** The table shows the results of all investigated biomarkers in urine corrected for urine creatinine concentrations and Cystatin C in serum of patients with SARS-CoV-2 infections and the comparisons of the concentrations in patients with or without AKI (left) and in patients with or without diabetes mellitus (right). Differences were evaluated by the Mann–Whitney U test and the results given in the table.

Biomarker Urine	No AKI Median (95% CI)	AKI Median (95% CI)	*p*-Value Mann-Whitney	Non-Diabetes Median (95% CI)	Diabetes Median (95% CI)	*p*-Value Mann-Whitney
HPLBII-P/Crea mg/mmol	0.016 (0.012–0.030) *n* = 32	0.041 (0.027–0.059 *n* = 41	*p* = 0.002	0.025 (0.016–0.036) *n* = 54	0.049 (0.029–0.117) *n* = 19	*p* = 0.008
HNL (763/8F)/Crea mg/mmol	2.0 (1.4–3.2) *n* = 33	3.3 (1.9–4.8) *n* = 41	Ns	2.1 (1.6–3.1) *n* = 54	5.9 (1.8–9.7) *n* = 20	*p* = 0.007
NGAL/Crea mg/mmol	4.1 (2.3–10.2) *n* = 19	7.8 (4.7–12.1) *n* = 30	Ns	4.8 (2.7–7.0) *n* = 36	27.6 (9.6–121) *n* = 13	*p* = 0.003
TIMP-2/Crea ng/mmol	0.45 (0.37–0.54) *n* = 19	0.50 (0.38–0.78) *n* = 30	Ns	0.46 (0.36–0.56) *n* = 36	0.53 (0.40–1.45) *n* = 13	Ns
KIM-1/Crea ng/mmol	0.32 (0.20–0.46) *n* = 33	0.28 (0.21–0.39) *n* = 41	Ns	0.31 (0.21–0.39) *n* = 55	0.23 (0.09–0.56) *n* = 19	Ns
Cystatin C/Crea mg/mmol	0.12 (0.06–0.16) *n* = 33	0.10 (0.08–0.15) *n* = 41	Ns	0.11 (0.08–0.13) *n* = 55	0.15 (0.05–0.34) *n* = 19	Ns
Albumin/Crea mg/mmol	12.6 (5.6–18.4) *n* = 33	13.6 (6.6–19.2) *n* = 41	Ns	12.6 (5.5–16.4) *n* = 55	14.6 (8.1–28.4) *n* = 19	Ns
**Biomarker** **Serum**	**No AKI** **Median (95% CI)**	**AKI** **Median (95% CI)**	***p*-Value** **Mann-Whitney**	**Non-Diabetes** **Median (95% CI)**	**Diabetes** **Median (95% CI)**	***p*-Value** **Mann-Whitney**
Cystatin C, mg/L	0.98 (0.80–1.05) *n* = 33	1.19 (1.11–1.35) *n* = 47	*p* = 0.0002	1.07 (0.99–1.12) *n* = 58	1.22 (1.06–1.80) *n* = 23	*p* = 0.055

Ns = non-significant.

## Data Availability

Data available on request due to restrictions e.g., privacy or ethical. The data presented in this study are available on request from the corresponding author. The data are not publicly available due to patent pending process.

## Supplementary Materials

The following supporting information can be downloaded at: https://www.mdpi.com/article/10.3390/jcm13092540/s1, Figure S1: The U-PLBII-P in males and females after urine creatinine correction; Figure S2: The correlations between urine albumin and urine HPLBII-P corrected for urine creatinine in the PIVUS cohort as separated by gender. The statistics are shown in the figure below.
