# Supplementary material for: The Human Phospholipase B-II Precursor (HPLBII-P) in Urine as a Novel Biomarker of Glomerular Activity in COVID-19 and Diabetes Mellitus
Source: J Clin Med. 2024 Apr 26;13(9):2540. doi: 10.3390/jcm13092540 (PMC11084804; doi:10.3390/jcm13092540)

Supplementary table and figures

Figure 1 supplement

The HPLBII-P concentrations in urine of patients with COVID-19 with and without AKI corrected for urine creatinine. Left panel shows the urine concentrations of HPLBII-P in relation to AKI stages and the right panel in relation to No AKI or AKI. Statistical differences were evaluated by Kruskal-Wallis ANOVA or by the Mann–Whitney U test as indicated, and the significances shown in the figures.

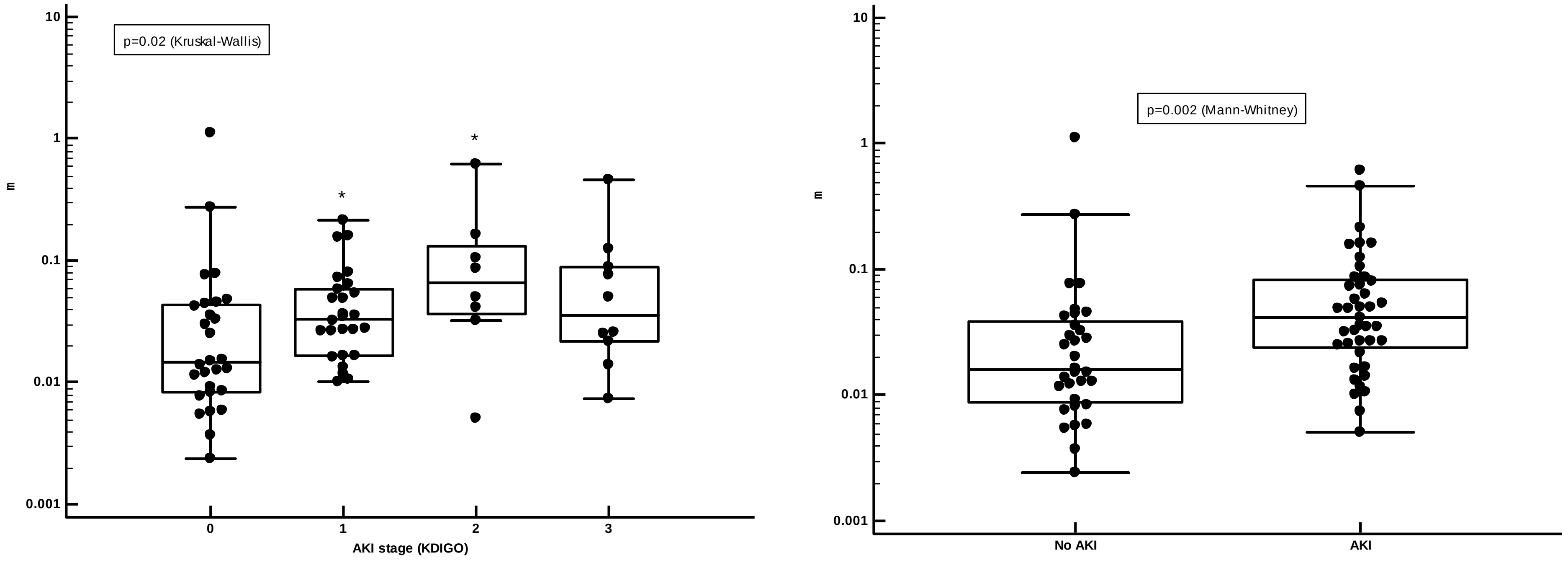

Figure 2 supplement

The urine concentrations of HPLBII-P in patients with COVID-19 with or without diabetes mellitus corrected for urine creatinine. The difference was evaluated by the Mann–Whitney U test and the significance given in the figure. The insert shows the results in the same cohorts but without AKI.

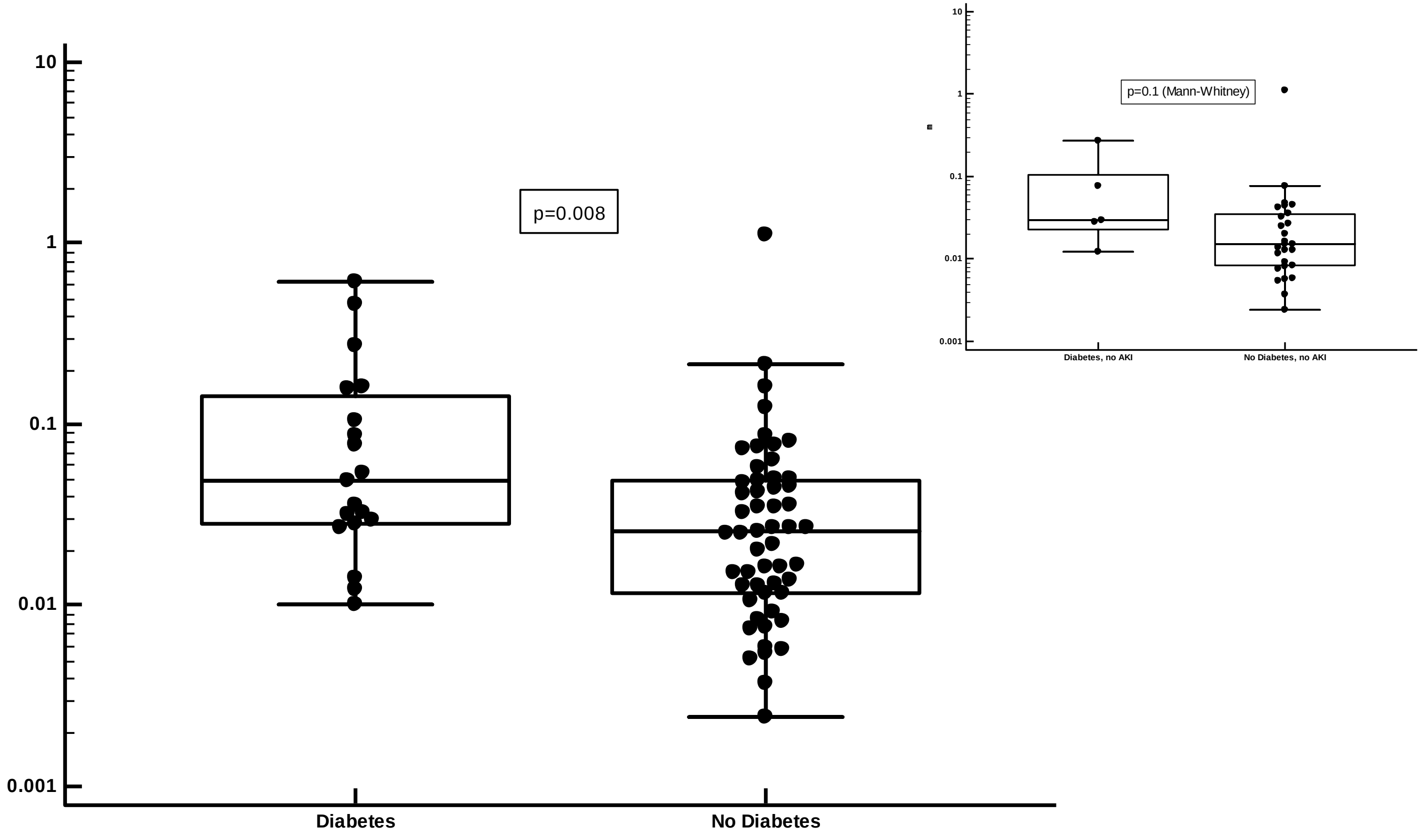

Supplement: Supplementary file 1 [file jcm-13-02540-s001.zip › jcm-2889993-supplementary.pdf]
